# Oncologic and Palliative Care in a Global Setting in the Twenty-First Century: The Patient, Family, and Oncologic Health Care Team

**DOI:** 10.1200/JGO.18.00076

**Published:** 2018-05-08

**Authors:** Manish A. Shah

**Affiliations:** New York-Presbyterian; Weill Cornell Medicine, New York, NY

Palliative care in oncology is a crucial issue and unmet need across the spectrum of cancer care and in countries and communities with varying degrees of resource allocation for cancer care. It is remarkable that the majority of patients newly diagnosed with cancer will ultimately die of their malignancy. Indeed, according to GLOBOCAN, 14 million patients were newly diagnosed with malignancy (excluding nonmelanoma skin cancer) in 2012, and approximately 8.2 million individuals died with malignancy in that same year (death rate of 58%).^1^ This issue is of particular importance in resource-limited countries in which the mortality rate for all cancers, excluding nonmelanoma skin cancer, is substantially higher at 66%.^1^ This is notable because the cancer burden in less-developed countries is greater, with more than 8 million cancer diagnoses. As might be expected, the fact that the mortality of an individual with a cancer diagnosis is significantly greater in lower- and middle-income countries highlights the need for access to palliative care in these areas, and for me, this is the most significant value of the Resource-Stratified Practice Guideline on Palliative Care in the Global Setting.^2^ Key issues this guideline addresses are the practical issue of how to deliver care in terms of palliative care models, the timing of palliative care, spiritual care, and pain management, among other salient and practical concerns when embarking on palliative treatment as it pertains to patients with cancer. However, beyond this obvious need, the guideline highlights another practical issue that can be observed across the globe, regardless of practice location and available resources: that of the oncology team.

Palliative care focuses on easing the symptoms of the disease. Although palliative care can be and is applied across medical care, it is particularly salient for patients with malignancy simply because of their high mortality rate. By relieving stress, pain, and suffering, palliative care can improve a patient’s quality of life and provide patients and care givers some peace of mind as they manage their terrible and often incurable illness. The early integration of palliative care into comprehensive oncologic care has been recommended by both the World Health Organization and ASCO.^3,4^ At issue is how best to deliver this care, particularly in resource-limited settings. Recently, a random assignment phase III study evaluated early palliative care intervention versus usual care, in which the palliative care intervention was led by palliative care nurses.^5^ The study by Vanbutsele et al^5^ involved significant components applicable to palliative care in resource-limited settings in which the recommendation for integration of palliative care involves a team approach and may involve primary health care providers, nurses, and community health care workers.^2^ In the study by Vanbutsele et al, patients were randomly assigned to early palliative care intervention, which typically started within 3 weeks of randomization. A key component of the study included training sessions conducted by oncologists to inform palliative care nurses about cancer treatments, anticipated adverse effects, and complications that might be expected early in the disease course. The palliative care interventions by these nurse specialists involved semi-structured, monthly palliative care consultations, monthly symptom assessments that used the Edmonton Symptom Assessment Scale, and participation of the palliative care nurses in weekly multidisciplinary oncology meetings.^5^ As in previous studies, overall quality of life 12 weeks after intervention was significantly improved with palliative care consultations. However, the most compelling aspect of this study was that the care was fully administered by palliative care nurses who were not necessarily familiar with the malignancy, its treatment, or anticipated adverse effects of treatment. This means that palliative care for patients with cancer, at least some aspects of it, can be provided by individuals who are part of the health care team available to the patient, even in the most basic settings, as suggested by the ASCO Resource-Stratified Practice Guideline on Palliative Care in the Global Setting.^2^

Is this team approach to patient care novel? Oncology and oncologic care, even in enhanced settings (perhaps particularly in enhanced settings), is more often provided using a team approach. Patients can often relate to their oncologist, but there may be other members of their oncologic team such as a nurse practitioner or physician assistant, oncology fellow, chemotherapy nurse, social worker, nutritionist, or others involved with their care. This emerging team approach raises the question, Are there times when an oncologist is necessary? For an oncologist, that question is self evident, but in the palliative care setting, that question was recently asked by Simon et al^6^ with regard to the patient’s understanding of his or her disease. In a large cohort study of 277 patients with late-stage cancer who had less than 6 months to live, patients were twice as likely to state that their disease was advanced if the oncologist was in the room when their scan was discussed compared with any other team member such as an oncology fellow, resident, or nurse practitioner. This is especially important because acceptance of palliative care depends on the patient accurately understanding the status of his or her illness and its outcome. Recent studies indicate that 38% of patients who are a median of 4 months from death acknowledged that they were terminally ill,^7^ but only 5% of terminally ill patients reported a complete understanding of their prognosis.^8^ Among the study cohort in the report by Simon et al,^6^ nearly 50% of the scans were reviewed by an oncologist only, whereas nearly 30% of the scans were reviewed by a nononcologist provider (eg, oncology fellow or physician extender). When an oncologist was present, patients were significantly more likely to understand that their disease was late stage.^6^ Oncologists who are present in the clinic to discuss scan results have patients who better understand the late stage of their disease and are thus better equipped to make informed end-of-life care choices.

Together, these studies highlight the realities of oncologic care in all settings. Patients with cancer have complicated illnesses and require multiple individuals to help with disease management. This team approach, if properly applied, can greatly help patients with their well-being as the team works to manage their illness. Some aspects of oncologic and palliative care, such as symptom assessment and management, can be managed by physician extenders and supporting members of the oncologic team. Other aspects of care, such as informing patients about their disease status, prognosis, and implications for ongoing care, are better conveyed by direct oncologist-patient interactions. The Resource-Stratified Practice Guideline on Palliative Care in the Global Setting has done a masterful job in helping frame the oncology care model along this spectrum. The guideline provides an excellent overview of all aspects of palliative care and creates a framework for how best to provide palliation in resource-limited settings.
